# Characterization of the complete mitochondrial genome of *Lysmata amboinensis* (Hippolytidae, Decapoda) and its phylogenetic analysis

**DOI:** 10.1080/23802359.2020.1842262

**Published:** 2020-12-24

**Authors:** Yan Wang, Ling Zeng, Jing Wen, Xuyan Li, Yafen Huang, Yulin Sun, Juan Zhao

**Affiliations:** a Department of Scientific Research, Lingnan Normal University, Zhanjiang, PR China; b Department of Chemistry, Lingnan Normal University, Zhanjiang, PR China; c Department of Biology, Lingnan Normal University, Zhanjiang, PR China

**Keywords:** *Lysmata amboinensis*, mitochondrial genome, phylogenetic analysis, Hippolytidae

## Abstract

The complete mitochondrial genome of *Lysmata amboinensis* was obtained and described in this study. This complete mitochondrial genome is 16,735 bp in length and consists of 13 protein-coding genes (PCGs), 2 ribosomal RNA genes (rRNA), and 22 transfer RNA genes (tRNA). A total of 23 genes were encoded by the heavy strand. The overall base composition of the heavy-strand was 31.68% A, 14.31% G, 21.65% C, and 32.36% T, with a high A + T content of 64.05%. The phylogenetic analysis suggested that hippolytidae shrimp may be considered as the polyphyletic taxon. These results are useful for understanding the phylogenetic relationships and evolution of Hippolytidae shrimp.

*Lysmata amboinensis* is one of the cleaner shrimps due to its specialized fish-cleaning behavior (Baeza et al. 2009). It is a hermaphroditic shrimp and widely distributed in a circum-tropical sea area, especially in corals and anemones (Debelius 2001). *Lysmata amboinensis* has a red dorsum with one white line. Because of their beautiful colors and their harmonious behaviour with other aquatic species, *L. amboinensis* became popular marine ornamental species in the world. The embryonic and larval development of *L. amboinensis* in laboratory condition was investigated by Zhang (2012). The mitochondrial genome is an effective tool for species identification, molecular taxonomy, and population genetic analyses (Galtier et al. 2009). Nowadays, only a few mitochondrial DNA sequences of *L. amboinensis* could be found in GenBank. Lack of genetic resources has hindered the conservation and utilization of *L. amboinensis.* In this study, the complete mitochondrial genome of *L. amboinensis* has been reported, which provides genomic data for phylogenetic and evolutionary investigations of *Lysmata* and Hippolytidae.

The specimen was collected from Shenzhen, Guangdong province, China (N22°35′, E114°31′) and deposited at the Zoological Herbarium, Lingnan Normal University (Acc. Number SC20200415-16). The muscle of *L. amboinensis* was fixed in 100% ethanol and stored at −20 °C. Approximately 30 mg of muscle tissue was used for mitochondrial DNA (mtDNA) extraction with TIANamp Marine Animals DNA Kit (Tiangen, Beijing, China) according to the manufacturer’s specification. MtDNA was sequenced using the Illumina Hiseq Sequencing System (Illumina Inc., San Diego, CA). The clean data were acquired and assembled by the SPAdes and PRICE (Bankevich et al. 2012). BLAST (http://www.ncbi.nlm.nih.gov/BLAST/), ORFs finder (https://www.ncbi.nlm.nih.gov/orffinder/) and MITO (Bernt et al. 2013) were used to identify and annotated protein-coding genes (PCGs). MITO (Bernt et al. 2013) was used to identify tRNA genes. A phylogenetic tree was constructed using MEGA version 6.0 software (Tamura et al. 2013).

The mitochondrial genome of *L. amboinensis* is 16,735 bp in length (GenBank accession number: MT762277), containing the typical set of 13 PCGs, 22 tRNA, and 2 rRNA genes. The heavy strand consists of 31.68% A, 14.31% G, 21.65% C, and 32.36% T, with a high A + T content of 64.05%. Of the 37 genes, 23 genes were encoded by the heavy strand and 14 genes including 4 protein-coding genes (PCGs), 2 ribosomal RNA genes (rRNA), and 8 transfer RNA genes (tRNA) were encoded by the light strand. Most PCGs had ATN as the start codon except ND5 initiated with GTG. Nine PCGs terminated with a complete stop codon, but four PCGs (*ND3*, *ND4*, *ND5*, and *Cytb*) had an incomplete stop codon (T––). The 16S and 12S rRNAs were 1323 bp (74.0% AT content) and 823 bp (75.1% AT content) in length, respectively. All tRNA genes ranged from 64 to 71 bp in size, and had the typical cloverleaf secondary structure. A non-coding region located between the 12S rRNA and tRNAIle genes was 918 bp in length.

Based on the complete 13 concatenated PCGs from 32 shrimps from GenBank database, a phylogenetic tree was constructed by the maximum likelihood (ML) method (Figure 1). *Lysmata amboinensis*, *Thor amboinensis*, and *Lebbus groenlandicus* belong to the Hippolytidae family. However, *L. amboinensis* was not clustered with the others, which suggested that Hippolytidae shrimp may be considered as the polyphyletic taxon. This newly reported genome of *L. amboinensis* will contribute to future phylogenetic studies and population genetic analyses for *L. amboinensis.*

**Figure 1.**
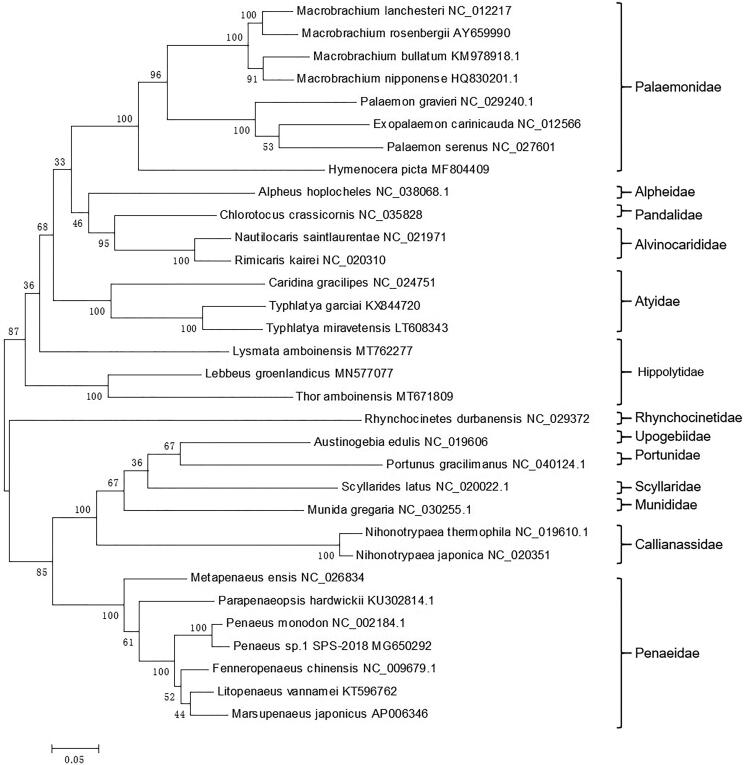
Phylogenetic tree of *L. amboinensis* and related species based on the maximum likelihood (ML) method.

## Data Availability

The data that support the findings of this study are openly available in NCBI at https://www.ncbi.nlm.nih.gov/, reference number MT762277.

## References

[CIT0001] Baeza JA, Schubart CD, Zillner P, Fuentes S, Bauer RT. 2009. Molecular phylogeny of shrimps from the genus Lysmata (Caridea: Hippolytidae): the evolutionary origins of protandric simultaneous hermaphroditism and social monogamy. Biol J Linn Soc. 96(2):415–424.

[CIT0002] Bankevich A, Nurk S, Antipov D, Gurevich AA, Dvorkin M, Kulikov AS, Lesin VM, Nikolenko SI, Pham S, Prjibelski AD, et al. 2012. SPAdes: a new genome assembly algorithm and its applications to single-cell sequencing. J Comput Biol. 19(5):455–477. 22506599 10.1089/cmb.2012.0021 PMC3342519

[CIT0003] Bernt M, Donath A, Jühling F, Externbrink F, Florentz C, Fritzsch G, Pütz J, Middendorf M, Stadler PF. 2013. MITOS: improved de novo metazoan mitochondrial genome annotation. Mol Phylogenet Evol. 69(2):313–319. 22982435 10.1016/j.ympev.2012.08.023

[CIT0004] Debelius H. 2001. Crustacea guide of the world. Frankfurt, Germany: IKAN–Unterwasserarchive.

[CIT0005] Galtier N, Nabholz B, Glemin S, Hurst GDD. 2009. Mitochondrial DNA as a marker of molecular diversity: a reappraisal. Mol Ecol. 18(22):4541–4550. 19821901 10.1111/j.1365-294X.2009.04380.x

[CIT0306] Tamura K, Stecher G, Peterson D, Filipski A, Kumar S. 2013. MEGA6: Molecular Evolutionary Genetics Analysis version 6.0. Mol Biol Evol. 30(12):2725–2729. 10.1093/molbev/mst197 PMC3840312 24132122

[CIT0006] Zhang YJ. 2012. Morphological observation of embryonic development and larval morphology of marine ornamental shrimp *Lysmata amboinensis.* Shanghai Ocean University I-II pp (abstract in English only).

